# Alcohol and airway calibre: Does motor or muscle depression contribute to increased likelihood of obstruction?

**DOI:** 10.1113/EP091055

**Published:** 2022-12-26

**Authors:** Michael N. Maxwell, Ken D. O'Halloran

**Affiliations:** ^1^ Department of Physiology School of Medicine College of Medicine & Health University College Cork Cork Ireland

**Keywords:** alcohol, genioglossus, motor unit, upper airway

1

The human pharynx is a compliant conduit for airflow, vulnerable at times to collapse, owing to suction forces generated during inspiration. Repeated obstructions of the upper airway occur during sleep, giving rise to obstructive sleep apnoea syndrome (OSAS), which is very common (Benjafield et al., 2019). Obstructive sleep apnoea syndrome is a debilitating condition associated with a broad portfolio of morbidities, including cardiometabolic and neurocognitive impairments. Untreated OSAS increases the risk of premature death.

Multiple factors contribute to airway vulnerability and propensity for arousal from sleep, resulting in a growing recognition of different OSAS endotypes. Additionally, lifestyle factors are important determinants of the propensity for airway occlusive events. Alcohol is a known depressant, and consumption worsens the severity of OSAS, measured as an increase in the number of obstructive events per hour of sleep. Studies in animals (Vecchio et al., 2010) and humans (Krol et al., 1984) have demonstrated that alcohol decreases tongue (genioglossus) EMG activity, revealing an inhibitory influence on motor control of upper airway muscles, which might relate to direct actions on cranial motor neurons innervating the tongue and/or depression of supramedullary excitatory inputs to cranial (hypoglossal) motor neurons. In this issue of *Experimental Physiology*, Avraam et al. (2023) describe, for the first time, the influence of acute alcohol ingestion on subpopulations of genioglossus motor units in healthy human participants, with an expectation that differential effects on subclasses of units would be revealed.

Young, healthy adult men and women were studied in a cross‐over study design. In one session, alcohol was consumed orally over 30 min at a dose that raised breath alcohol concentration to 0.07%, equivalent to about four standard drinks. A placebo trial was also conducted, although participants easily differentiated the two trials. Nevertheless, control measurements with 0.00% breath alcohol concentration were achieved, allowing paired comparisons within individuals. Electroencephalogram and masseter EMG recordings confirmed that participants remained awake during motor unit recording sessions. Participants were instrumented with four intramuscular monopolar genioglossal electrodes. A composite of the four recordings allowed for the estimation of global genioglossal activity, whereas spike sorting was used to discriminate motor units from different tongue regions, with motor units classified into standard predetermined categories related to the respiratory cycle.

Surprisingly, Avraam et al. (2023) found only modest, non‐significant effects of alcohol consumption on global genioglossal EMG activity. Moreover, although a decrease in inspiratory motor unit activity was expected, the number of active phasic inspiratory motor units was found to increase after alcohol consumption, which, it is argued, might have arisen from a potential increase in airway resistance attributable to alcohol‐related nasal congestion. The total number of active units and the peak discharge frequencies of units did not differ between conditions. Interestingly, the general lack of effect of alcohol on motor unit behaviour was also noted during repeated hypoxic exposures and subsequent normoxic periods wherein a lasting after‐discharge was evident. As such, alcohol was shown to have no effect on the capacity to raise genioglossal activity in response to chemoreceptor‐dependent challenge and the associated short‐term potentiation of activity after removal of the hypoxic stimulus.

It is difficult to reconcile these findings with previously published literature. One argument is that assessments were performed during wakefulness, and therefore the outcomes might differ during sleep states. Certainly, state‐dependent changes in neuromodulatory facilitation of hypoglossal motor output are recognized. Indeed, a greater appreciation of these mechanisms has led to the development of pharmacotherapies for OSAS (Lim et al., 2021). Loss of a wakefulness drive in sleep contributes to hypotonia of upper airway muscles and decreased airway calibre and/or stiffness. Therefore, it would certainly be interesting to explore the effects of alcohol on genioglossus EMG and motor unit behaviour throughout sleep–wake cycles. However, to attribute the lack of effect of alcohol on genioglossal motor unit activity to the prevailing wakefulness drive is to suggest that alcohol has no discernible effect on that drive, and yet it is reported in rats that the alcohol‐induced decrease in genioglossus activity is likely to relate to a reduction in this excitatory input, more so than direct inhibitory effects at the level of the XIIth nucleus (Vecchio et al., 2010). It is unclear whether genioglossus muscle force was reduced after alcohol consumption in participants, independent of potential influences on motor control. Acute ethanol disrupts intracellular calcium flux in skeletal muscle (Cófan et al., 2000). Direct assessment of the force‐generating capacity of the tongue would reveal whether alcohol ingestion results in muscle weakening, which could be a factor contributing to an increased incidence of obstructive events, perhaps especially in people with OSAS who have airways vulnerable to collapse.

Therefore, studies of the effects of alcohol on genioglossus EMG and motor unit activity throughout the sleep–wake cycle in healthy participants and people with OSAS are warranted. Notwithstanding the findings of Avraam et al. (2023), evidence that alcohol consumption worsens the severity of OSAS counsels that a ‘nightcap’ is contraindicated in people at risk of obstructive airway events and is probably best avoided even by others!

## AUTHOR CONTRIBUTIONS

Both authors have read and approved the final version of this manuscript and agree to be accountable for all aspects of the work in ensuring that questions related to the accuracy or integrity of any part of the work are appropriately investigated and resolved. All persons designated as authors qualify for authorship, and all those who qualify for authorship are listed.

## CONFLICT OF INTEREST

None declared.

## FUNDING INFORMATION

None.
